# Natural transformation of *Vibrio cholerae *as a tool - Optimizing the procedure

**DOI:** 10.1186/1471-2180-10-155

**Published:** 2010-05-28

**Authors:** Rasmus L Marvig, Melanie Blokesch

**Affiliations:** 1 Global Health Institute, Ecole Polytechnique Fédérale de Lausanne (EPFL), Lausanne, Switzerland

## Abstract

**Background:**

*Vibrio cholerae *gains natural competence upon growth on chitin. This allows the organism to take up free DNA from the environment and to incorporate it into its genome by homologous recombination.

**Results:**

Making use of this developmental program in order to use it as a tool to genetically manipulate *V. cholerae *and potentially also others *Vibrio *species was envisaged. Therefore, we re-investigated the experimental design for natural transformation of *V. cholerae *and tested different donor DNA fragments with respect to their source (genomic versus PCR-derived), quantity, and homologous flanking regions. Furthermore, we simplified the procedure in terms of the chitin source used as inducer of natural competence and the composition of the growth medium.

**Conclusions:**

The current study allows us to recommend a standard protocol to genetically manipulate *V. cholerae *using commercially available sources of chitin and minimal medium, respectively, as well as PCR-derived donor DNA as transforming material.

## Background

*Vibrio cholerae *is a human pathogen. However, "cholera bacilli" are also normal members of aquatic environments where they live in association with the chitinous exoskeleton of zooplankton (e.g. copepods) and their molts [1].

The genome sequence of *V. cholerae *[2] as well as comparative genomic hybridization experiments have revealed evidence for gene acquisition via horizontal gene transfer [3-6]. Furthermore, analysis of the genome of another aquatic *Vibrio*, *Vibrio vulnificus *YJ016, revealed a high degree of sequence identity to non-*Vibrio *bacteria, which again led to the conclusion that these sequences were horizontally acquired [7].

A recent study showed that *V. cholerae *gains natural competence upon growth on chitin surfaces [8]. Natural competence enables these bacteria to take up free DNA from the environment in order to incorporate it into their genome. Blokesch and Schoolnik demonstrated that the whole O1 specific antigen cluster (size of ~32 kb) of *V. cholerae *O1 El Tor can be exchanged either by the O37- (size of ~23 kb) or by the O139-specific antigen cluster (size of ~42 kb) by means of chitin-induced natural competence [9]. Following this first publication, we and others demonstrated that indeed different parts of the genome can be horizontally transferred in this manner including parts of the *Vibrio *pathogenicity island 2 (VPI-2) and the *Vibrio *seventh pandemic islands (VSP-I and VSP-II), respectively (Blokesch and Schoolnik, unpublished data), the cholera toxin prophage [10] and clusters encoding metabolic pathways [6].

A recent study by Gulig *et al*. confirmed our notion that natural competence might be a common feature of different *Vibrio *species [11]. In their study *Vibrio vulnificus*, another chitinolytic aquatic *Vibrio *species, was shown to be naturally transformable upon exposure to chitin surfaces following the crab-shell associated transformation protocol established earlier for *V. cholerae *[8]. This study as well as frequent inquiries from other researchers about chitin-induced natural transformation encouraged us to optimize and simplify the chitin-induced natural competence protocol in order to make in amenable as a tool to the *Vibrio *research community.

## Methods

### Bacterial strains

The *Vibrio cholerae *strains used in this study were *V. cholerae *O1 El Tor A1552 [12] and its nuclease minus derivative A1552Δdns [13]. Strain A1552-LacZ-Kan harboring a Kanamycin resistance cassette (aminoglycoside 3'-phosphotransferase; *aph*) within the *lacZ *gene of *V. cholerae *O1 El Tor strain A1552 (this study) was used to provide donor genomic DNA (gDNA) for the transformation experiments and as template in PCR reactions, respectively.

### Media and growth conditions

For transformation experiments *V. cholerae *cultures were grown either in defined artificial seawater medium (DASW) as described [8] or in M9 medium [14] supplemented with MgSO_4 _and CaCl_2 _as recommended by the manufacturer (Sigma). Additional NaCl, HEPES, MgSO_4 _and CaCl_2_, was added as indicated in the text. Selection was performed on LB agar plates [15] containing Kanamycin at a concentration of 75 μg ml^-1^. Total colony forming units (CFUs) were quantified on plain LB agar plates.

### Chitin-induced natural transformation

Natural transformation experiments on crab shell fragments were performed as described [8,9]. Variations thereof were used in order to test different chitin/chitin derivative sources: *V. cholerae *A1552 cells were grown at 30°C until an OD_600 _of approximately 0.5, washed and resuspended in DASW or M9 medium. Autoclaved chitin flakes, chitin powder or chitosan (50-80 mg each) were subsequently inoculated with 0.5 ml washed bacterial culture plus 0.5 ml fresh medium, mixed thoroughly and incubated at 30°C for 16-20 hours. After exchange of the medium (except where indicated) donor DNA was added as transforming material. The DNA was either gDNA of strain A1552-LacZ-Kan (positive control) or PCR-derived DNA as explained in the text. Cells were further incubated for either 2 hours (expedite protocol) or 24 hours (standard protocol), respectively, and subsequently detached from the chitin surface by vigorously vortexing for 30 sec. Transformants were selected on LB + Kanamycin (75 μg ml^-1^) plates and transformation frequencies were scored as number of Kanamycin-resistant CFUs/total number of CFUs. Chitin and chitin derivatives used in this study: Chitosan (Fluka; cat. # 448869), Chitin flakes (Sigma; cat. #C9213), Chitin powder (Sigma; cat. # C7170) and Dungeness crab shells (Fisherman's Wharf, San Francisco, CA).

### Polymerase chain reactions

PCR fragments were acquired using the oligonucleotides listed in Table 1 and following the protocol recommended by the manufacturer of the polymerase (Expand High Fidelity system, Roche). Genomic DNA of strain A1552-LacZ-Kan (this study) and plasmid pBR-lacZ-Kan-lacZ, respectively, served as template. The latter plasmid was constructed by ligating the PCR-derived *lacZ*-flanked Kanamycin cassette (aminoglycoside 3'-phosphotransferase gene; *aph*) of strain A1552-LacZ-Kan (primers Nhe-lacZ-start and LacZ-end-SalI; Table 1) into the *Eco*RV-digested plasmid pBR322 [16].

**Table 1:** Oligonucleotides used in this study

Primer name	Sequence
*Nhe*I-lacZ-start	5'-PCGCGCTAGCAAAGGCGTTATTGGCTTGTTGC-3'

LacZ-end-*Sal*I	5'-PCGCGTCGACGCTTTCACACGTAAGGTGAGC-3'

Tfm-II-1000	5'-CGGGAAGCTAGAGTAAGTAGTTCG-3'

Tfm-II+1000	5'-CGTTCCATGTGCTCGCCGAGGCG-3'

Tfm-II-gDNA-1000	5'-AAGCTTCCTGCTTGGAAGAAATGGC-3

Tfm-II-gDNA+1000	5'-CGGTGTATCTGTGGCAACGGTTTC-3'

Tfm-II-2000	5'-CCCCCCTGACGAGCATCACAAAAATCG-3'

Tfm-II+2000	5'-CTGACGCGCCCTGACGGGCTTGTCTGC-3'

Tfm-II-gDNA-2000	5'-GAAACCGACGAAGGTGTGTTGATC-3'

Tfm-II-gDNA+2000	5'-CGCAACCGGATTGGTGCGCTATTTTGGC-3'

KanR-500flank-up	5'-GCGCTTTATCAACACGCTGAATTGC-3'

KanR-500flank-down	5'-ACGCGAAGATCGTCACATTCCACAC-3'

KanR-250flank-up	5'-TGCTTGATGAAGATGGCGCGCCG-3'

KanR-250flank-down	5'-CATCTTGCTGCCATTGAGGCAGCG-3'

KanR-100flank-up	5'-ATGTGATGGATGAAGCAAGCATGCG-3'

KanR-100flank-down	5'-ATTCATGCTCTGGCAACATTGGCAGC-3'

### Statistics

Statistical analysis concerning difference between two means was done using the Student's *t *test. A 2^4 ^factorial design was performed to assess the effects of growth medium supplementation on transformation frequencies. Statistical analyses of the data was done using JMP^® ^software (SAS Institute Inc., Cary, USA).

## Results

Introducing DNA into a bacterial chromosome in order to genetically manipulate it can be challenging. Learning from the environmental lifestyle of some bacteria might give us new insights into their modes of DNA uptake/transfer. Following this strategy it was recently discovered that *V. cholerae *acquires natural competence upon growth on chitin [8], a feature that is shared by another chitin-colonizer, *V. vulnificus *[11]. Using this natural transformability as a tool for genetic manipulations is a logical consequence. We therefore decided to establish a simplified natural transformation protocol.

### The extracellular nuclease Dns partially inhibits natural transformation of wild-type cells

**Figure 1:**
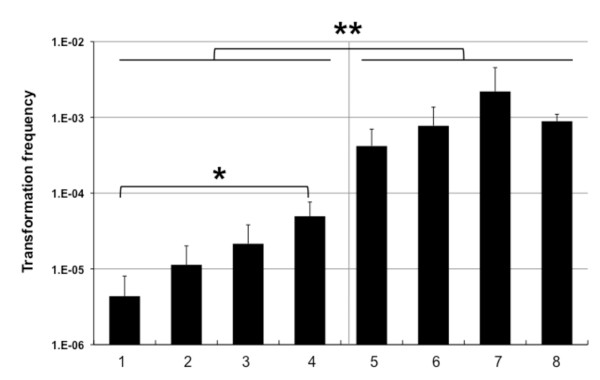
**The amount of donor DNA determines transformation frequencies**. *V. cholerae *strains A1552 (WT; lanes 1-4) and A1552Δdns (5-8), respectively, were naturally transformed on crab shell fragments with increasing amounts of donor genomic DNA (gDNA) of strain A1552-LacZ-Kan. Amounts of donor gDNA provided: 0.2 μg (lanes 1 and 5), 2 μg (lanes 2 and 6), 20 μg (lanes 3 and 7) and 200 μg (lanes 4 and 8). Average of at least three independent experiments. Student's *t *test: * statistically significant difference between lowest and highest amount of donor gDNA (*p *< 0.05); ** statistically significant difference between wild-type and nuclease minus strain (*p *< 0.01).

In the previous protocol for chitin-induced transformation of *Vibrio *2 μg of donor genomic DNA (gDNA) were provided [8]. We tested whether DNA quantity influences the transformation frequency by adding increasing amounts of donor gDNA ranging over fours orders of magnitude (0.2 μg until 200 μg; Fig. 1). We observed increasing frequencies (Fig. 1, lanes 1 to 4) with a statistically significant difference between the addition of 0.2 ug (lane 1) and 200 ug (lane 4), respectively.

The fact that higher amounts of donor DNA give rise to higher transformation frequencies can have two not mutually exclusive reasons: 1) The amount of DNA is at sub-saturation level and thus the more DNA is provided the more DNA is taken up and might get homologously recombined into the chromosome; 2) The donor DNA might be degraded before uptake, e.g. outside of the bacteria. To follow up on the latter hypothesis we repeated the experiment using an extracellular nuclease minus strain (A1552Δdns; [13]), which was shown to be hypertransformable [13]. Under these conditions we did not observe any statistically significant change in transformation frequency by adding increasing amounts of donor gDNA (Fig. 1, lanes 5 to 8). Thus, the amount of donor gDNA is saturating for this strain with respect to the transformation process itself. This allow us to conclude that in the case of the wild-type strain (Fig. 1, lanes 1 to 4) part of the donor DNA might be degraded before uptake, e.g. outside of the bacteria, so that excess of DNA helps to protect transforming DNA against degradation.

### PCR fragments can be used as donor DNA for natural transformation

Moving genomic fragments, including selective marker(s), from one strain to another is certainly doable by this method. Nevertheless, to genetically manipulate new strains with the aid of PCR-derived constructs is more desirable. One possibility to do so is to amplify the flanking genomic regions, contemplated for an antibiotic marker insertion by PCR, as well as the antibiotic resistance cassette itself and combining them in a second round of PCR reaction. This has been done successfully resulting in the integration of a Kanamycin resistance cassette (*aph*) into the O37 antigen region of strain ATCC25873 by chitin-induced natural transformation [9]. In contrast to this, the study of Gulig *et al*. reported very low efficiency using PCR-derived donor DNA for *V. vulnificus *[11].

**Figure 2:**
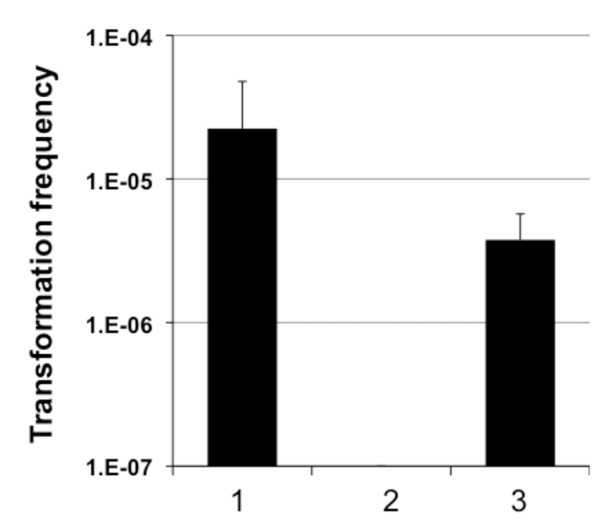
**PCR fragments can serve as donor DNA**. *V. cholerae *wild-type strain A1552 was induced for natural competence on crab shell fragments and scored for its transformation frequency (Y-axis). Provided donor DNA was derived either from strain A1552-LacZ-Kan as a positive control (2 μg gDNA; lane 1), or from a PCR reaction according to IV in Fig. 3A. PCR-derived DNA was purified before administered to the bacteria (lane 3; 200 ng). The negative control, with no donor DNA provided, is shown in lane 2. Average of at least three independent experiments.

To follow up on this we PCR-amplified approximately 3700 bp of DNA comprising the Kanamycin resistance gene aminoglycoside 3'-phosphotransferase (*aph*) using plasmid pBR-lacZ-Kan-lacZ as template. This plasmid is a derivative of pBR322 [16] with a *lacZ*'-Kan-'*lacZ *insert in the multiple cloning site (for details see Methods; the *lacZ *flanking regions were derived from *V. cholerae *O1 El Tor). The resulting PCR fragment was given to competent wild-type *V. cholerae *cells and the transformation frequency in comparison to a control using gDNA was determined (Fig. 2, lanes 1 and 3). As shown in Fig. 2 the PCR fragments were indeed able to serve as transforming material and resulted in a 10-fold lower transformation frequency than the gDNA control. No spontaneous Kanamycin-resistant colonies appeared in the absence of any donor DNA (Fig. 2, lane 2).

The next question we wanted to address was why the transformation frequency using PCR-derived donor DNA is low compared to the provision of gDNA. We considered two main reasons: Degradation and/or reduced homologous recombination due to the shorter PCR fragments.

### Contribution of the flanking regions towards natural transformation

**Figure 3:**
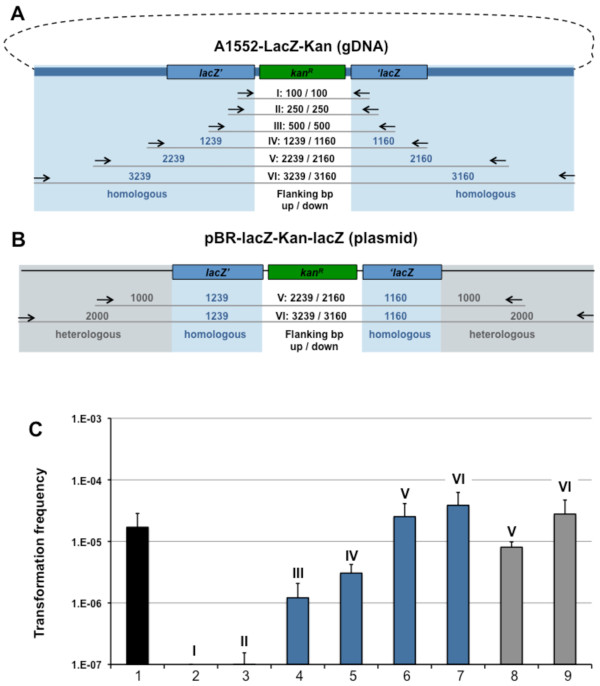
**PCR-derived donor DNA with various lengths of homologous and heterologous flanking regions**. Panel A: PCR-derived fragments using genomic DNA of strain A1552-LacZ-Kan as template. Priming region of oligonucleotides (Table 1) are indicated. Primer combinations used: (I) KanR-100-flank-up & KanR-100-flank-down; (II) KanR-250-flank-up & KanR-250-flank-down; (III) KanR-500-flank-up & KanR-500-flank-down; (IV) NheI-lacZ-start & LacZ-end-SalI; (V) Tfm-II-gDNA-1000 & Tfm-II-gDNA+1000; (VI) Tfm-II-gDNA-2000 & Tfm-II-gDNA+2000. Panel B: Plasmid pBR-lacZ-Kan-lacZ was used as PCR template and is shown in a linearized fashion. The following primer pairs were used: (V) Tfm-II-1000 & Tfm-II+1000; (VI) Tfm-II-2000 & Tfm-II+2000. Sizes of the up- and downstream flanking regions with respect to the Kanamycin resistance cassette are indicated in the middle. Blue shading: region homologous to recipient strain (A1552; wild-type); grey shading: heterologous region. Panel C: *V. cholerae *wild-type strain A1552 was naturally transformed using the crab-shell transformation protocol and PCR-derived DNA according to Panels A and B. Transformation frequencies are shown on the Y-axis using either 2 ug gDNA of strain A1552-LacZ-Kan as positive control (lane 1; black) or 200 ng of PCR-derived DNA with varying length of the homologous (in blue; lane 2 to 7; according to Panel A) or homologous + heterologous (in grey; lane 8 and 9; according to Panel B) flanking region. Length of Kan^*R*^-flanking DNA: lane 2: 100 bp, lane 3: 250 bp; lane 4: 500 bp; lanes 5: ~1000 bp; lane 6 and 8: ~2000 bp; lane 7 and 9: ~3000 bp. Average of at least three independent experiments.

To further investigate what exactly influences natural transformability we investigated the effect of the length of flaking regions. Using the primers listed in Table 1 we amplified PCR fragments possessing between 100 bp and 3000 bp flanking regions up- and downstream of the Kanamycin cassette (*aph *gene; Fig. 3 for details). Genomic DNA of strain A1552-LacZ-Kan (Fig. 3A) or plasmid pBR-lacZ-Kan-LacZ (Fig. 3B) served as template and the resulting PCR fragments were tested for their ability to serve as transforming material (Fig. 3C). Using this strategy we were able to determine a required length of the flanking regions as being ≥ 500 bp in order to acquire transformants reproducibly (Fig. 3C, lane 4 to 7). Beyond a flanking-region-length of 2000 bp no substantial increase in transformation frequency occurred (Fig. 3C, lane 6 versus 7). By using plasmid pBR-lacZ-Kan-LacZ as template we acquired PCR fragments with mixed flaking regions: homologous DNA close to the antibiotic resistance cassette and heterologous DNA up- and downstream thereof (Fig. 3B, fragments V and VI). These homologous/heterologous flanks also increased the transformation frequency (Fig. 3C, lanes 8 and 9) when compared to fragments containing only the homologous part (Fig. 3C, lane 5).

### Changing the source of chitin to simplify the natural transformation protocol

**Figure 4:**
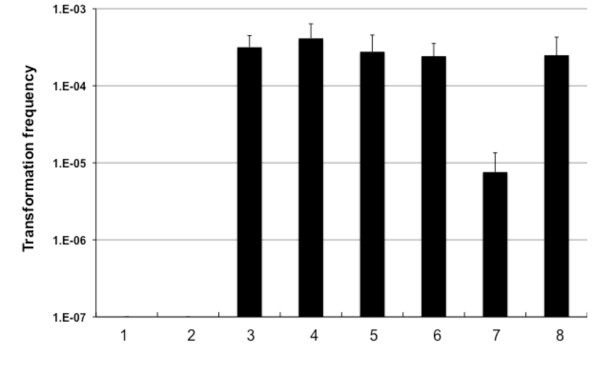
**Induction of natural competence by different chitin sources**. Different chitin sources and chitin derivatives were tested for their ability to induce natural competence in *V. cholerae *A1552. Lanes 1 and 2: chitosan; lanes 3 and 4: chitin flakes; lanes 5 and 6: chitin powder; lanes 7 and 8: crab-shell fragments (approx. 1 cm^2^). The medium was not changed at the time of donor DNA (2 ug LacZ-Kan gDNA) addition for all odd lanes but for all even lanes. Average of four independent experiments.

To uniform the chitin substrate and make it available to researcher without access to crab shells we tested other forms of chitin or chitin-derivatives as inducer of natural competence (Fig. 4). Whereas chitosan, a deacetylated form of chitin, did not result in any detectable transformants (Fig. 4, lane 2), the other chitin sources (chitin flakes, lane 4; chitin powder, lane 6) worked very well and resulted in comparable transformation frequencies as in the case of crab-shells (Fig. 4, lane 8).

We also tested another variation from the standard transformation protocol using these different chitin sources (Fig. 4, lanes 1, 3, 5 and 7): after culturing the bacteria for 16 hours the surrounding medium was NOT exchanged; instead donor DNA was directly added (see Methods). This resulted in no difference in the case of chitin flakes and chitin powder as substrate (Fig. 4, lanes 3 and 5) in contrast to a 30-fold drop of transformation frequency using the crab shell protocol (Fig. 4, lane 7).

### Expedite protocol for natural transformation

As the experiments on chitin flakes did not require exchange of the growth medium we hypothesized that high cell densities were reached earlier. This would result in earlier down-regulation of nuclease expression and earlier induction of competence. Therefore we established an expedite protocol: Wild-type bacteria were grown on chitin flakes for 16 hours; at that time new medium and 2 μg of donor gDNA was provided and incubated statically for two hours. Cells were released by vortexing, plated and scored for CFUs on selective and plain medium, respectively. The average transformation frequency from four independent experiments was 5.0 × 10^-4 ^(SD 3.4 × 10^-4^) and thus comparable to the two days experimental procedure described earlier (3.17 × 10^-4^; see Fig. 4; lane 3).

### Using supplemented M9 minimal medium allows natural transformation to occur

As last component of the natural transformation procedure we were eager to simplify the composition of the growth medium. The initial protocol to naturally transform *V. cholerae *utilized defined artificial seawater medium (DASW) [8,9]. This medium has twelve different components and several of them are not present by default in every laboratory. In contrast, M9 minimal medium salts are commonly used. We compared the composition of standard M9 and DASW medium [8,14] (Table 2) and further concentrated on the contribution of four major factors towards natural transformability: NaCl, HEPES, MgSO_4 _and CaCl_2_.

**Table 2:** Comparison of M9 medium and defined artificial seawater medium (DASW) with respect to four main components tested

Component	M9 medium*	Enriched M9 medium (this study)	**DASW**^#^
NaCl	9 mM	259 mM	234 mM

HEPES	0 mM	50 mM	50 mM

MgSO^4^	2 mM	32 mM	27.5 mM

CaCl^2^	0.1 mM	5.1 mM	4.95 mM

* Sigma; supplemented as recommended by the manufacturer

^# ^As published [8,9]

**Figure 5:**
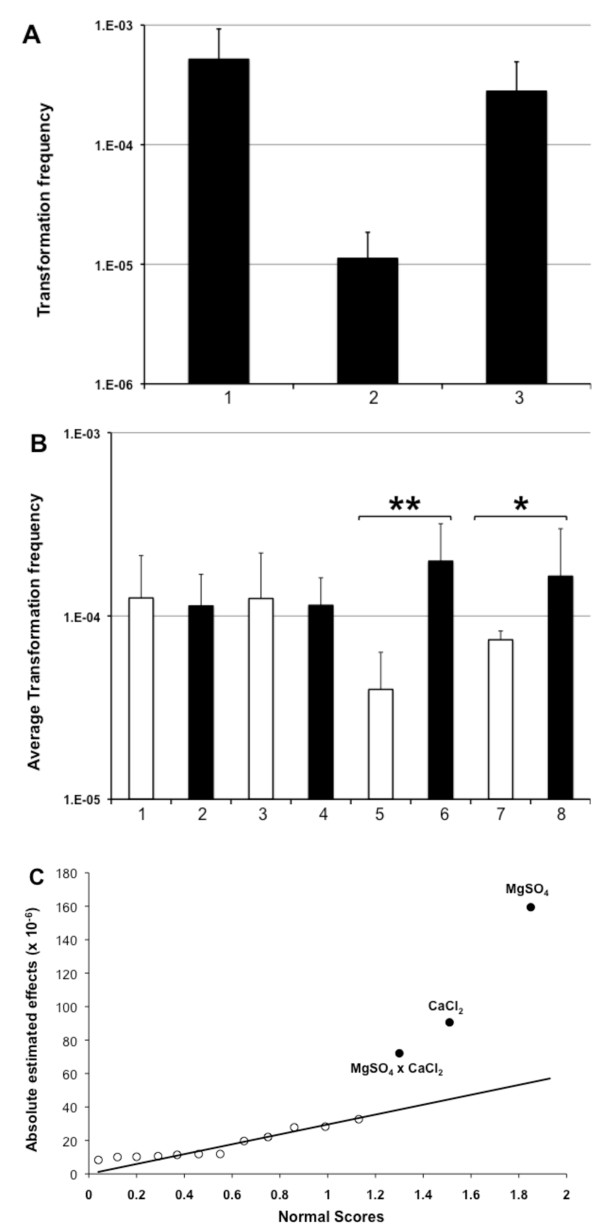
**Optimizing M9 minimal medium for chitin-induced natural transformation**. *V. cholerae *A1552 was induced for natural competence by growth on chitin flakes. Panel A: Transformation frequencies (y-axis) using the expedite transformation protocol and 2 ug of gDNA of strain A1552-LacZ-Kan as transforming material. The medium used was DASW (lane 1), standard M9 medium (lane 2), and MgSO_4 _/CaCl_2 _enriched M9 medium (lane 3; see text for details), respectively. Average of at least three independent experiments. Panel B: Commercially available M9 medium was used as base and alternated with respect to NaCl, HEPES, MgSO_4 _and CaCl_2_. White columns represent average transformation frequencies of all low concentration samples (lane 1: 9 mM NaCl; lane 3: 0 mM HEPES; lane 5: 2 mM MgSO_4_; lane 7: 0.1 mM CaCl_2_) comparable to standard ingredients of M9 minimal medium. Black columns represent average transformation frequencies of high concentration samples mimicking DASW concentrations (lane 2: 259 mM NaCl; lane 4: 50 mM HEPES; lane 6: 32 mM MgSO_4_; lane 8: 5.1 mM CaCl_2_). Statistically significant differences are indicated by asterisks (*p < 0.05; **p < 0.01). Panel C: Magnitude of main effects and interactions of factors influencing natural transformation. Half-normal plot of the absolute estimated values (Y-axis) versus their positive normal score (X-axis) are shown as white circles. Black circles indicate statistically significant effects due to addition of MgSO_4_, CaCl_2 _as well as both together (MgSO_4 _× CaCl_2_).

The effects of all four factors were evaluated using a replicated 2^4 ^full factorial design (Fig. 5). This required a replication of 16 experiments (combinations of low versus high concentration of each factor), which we ran in four independent blocks (eight runs per block) following the expedite protocol described above. The same experiment using DASW and standard M9 medium served as positive and negative control, respectively (Fig. 5A).

As can be seen in Fig. 5B there was no significant difference between low and high concentrations of NaCl (lane 1 versus 2). The presence/absence of HEPES was also of no importance (lanes 3 and 4). However, the addition of MgSO_4 _and CaCl_2_, respectively, turned out to be significant (lanes 5 versus 6 and 7 versus 8).

Looking at a half-normal plot (Fig. 5C) of the ordered factor effects (main effects and interactions; Y-axis) plotted against their positive normal scores (X-axis) helped us to indicate the most important effects [17]. Any large estimated effects (Fig. 5C, closed circles) are located above the straight-line pattern formed by the small estimated effects (Fig. 5C, open circles). We recognized that the addition of MgSO_4 _or CaCl_2 _as well as both components in concert had positive effects on transformation frequencies (Fig. 5C).

We therefore recommend using M9 minimal salts supplemented with MgSO_4 _and CaCl_2 _to a final concentration of 32 mM and 5 mM, respectively (Fig. 5A, lane 3).

## Discussion

Chitin-induced natural transformation enables *Vibrio cholerae *to acquire novel genes thereby evolving new traits, which render the bacterium better adapted to the environment or more pathogenic to man [8]. This needs further emphasis after a recent study by Blokesch and Schoolnik [9]: these authors showed that the O-antigen region can be transferred between different *V. cholerae *strains by means of chitin-induced natural transformation thereby rendering the recipient insensitive to certain O-antigen-specific bacteriophages (environmental benefit). This also provides a potential explanation for the devastating occurrence of the O139 serogroup in 1992, which infected persons previously immune to *V. cholerae *O1 El Tor [18] (more pathogenic for man).

A more recent contribution by the groups of G. Balakrish Nair, John Mekalanos and Shah M. Faruque in PNAS nicely confirmed what was hypothesized before, namely that transformation, in principle, can "mediate the transfer of fragments from any part of the genome" [9]. In this study Udden *et al*. were able to move the classical type cholera toxin prophage (CTX_class_) between different strains using chitin-induced natural transformation as mode of horizontal gene transfer [10]. As the donor O141 strain was unable to produce CTX_class _phage particles the DNA region was not transferable by phage transduction [10]. Thus, natural transformation might also contribute to the dispersal of the CTX prophage among different *V. cholerae *strains.

The presented study takes advantage of the natural competence program and describes an optimized procedure to use natural competence as a common tool for the manipulation of *Vibrio *genomes. As Gulig *et al*. recently demonstrated that also other aquatic *Vibrio *species acquire natural competence upon growth on chitin surfaces [11] this method might be applicable to several *Vibrio *species. In this particular publication, the authors also used PCR-derived donor DNA though transformants were often undetectable [11]. PCR-derived donor DNA was used successfully as transforming material by Blokesch and Schoolnik in a report published two years earlier [9] as well as by Udden *et al*. in 2008 [10]. In this present study, we showed that PCR-derived DNA could indeed serve as transforming material. Nonetheless, several other aspects needed to be optimized in order to adapt chitin-induced natural transformation as a standard protocol for manipulating *Vibrio *genomes. The major points addressed were: the quantity and quality of the donor DNA; the chitin source; and the composition of the medium.

We showed that donor DNA is readily degraded by the extracellular nuclease Dns [13] and that a higher amount of donor DNA can partly compensate for this (Fig. 1). Otherwise the usage of nuclease negative strains as recipients is recommended in case this does not interfere with consecutive experiments.

Also the source of the donor DNA turned out to be rather important: in Fig. 2 we compared PCR-derived versus genomic DNA. It appeared as if the transformation frequency was only one order of magnitude lower for PCR-derived donor DNA (200 ng; Fig. 2, lane 3) than for gDNA (2 μg; Fig. 2, lane 1). Though one has to consider that the amplified PCR fragment represents only 1/1000^th ^of the full *V. cholerae *genome. Thus the PCR-fragment was provided in 100-fold molar excess. But as PCR-fragments can be acquired in large amounts this might not be an unconquerable problem.

Several reasons could cause this relative low frequency of transformation, including DNA restriction/modification systems, increased sensitivity to degradation of the small DNA pieces and lack of homologous regions required for recombination. The group of Wilfried Wackernagel showed for another naturally competent bacterium, *Acinetobacter calcoaceticus*, that equal transformation efficiencies were scored no matter whether the donor DNA was isolated from *E. coli *or *A. calcoaceticus *itself. The authors concluded that restriction/modification systems are not involved in the natural transformation process [19]. In the case of *V. cholerae *we cannot exclude any contribution of restriction/modification systems. However, we did not observe any significant difference with respect to transformation frequencies using either unmodified PCR fragments or linearized plasmids containing the same flanking region as donor DNA (data not shown). Furthermore, in the case of natural transformation special mechanisms are involved in the protection of the incoming DNA. One such candidate is DprA, a protein that, in *Streptococcus*, binds single stranded DNA once it reaches the cytoplasm and prevents it from degradation [20,21]. The gene for *V. cholerae*'s DprA homologous is induced upon growth on chitin [22] and essential for natural transformation. Consequently, *V. cholerae *might employ the same strategy, e.g. the protection of the incoming DNA by DprA, which then guides it to RecA for homologous recombination.

In terms of homologous recombination we compared donor DNA with flanking region that were either homologous to the recipient's genome (Fig. 3A, C) or a mixture of homologous and heterologous (Fig. 3B, C). It turned out that homologous flanking regions do bear an advantage over non-homologous regions (Fig. 3, lane 6 versus lane 8) though by further increasing the length of the flanks the difference in transformation frequency was negligible (Fig. 3, lane 7 versus lane 9). With respect to the length of the flanking region we observed an approximately 20-fold increase in transformation frequency from 500 bp flanking regions on both ends (Fig. 3C, lane 4) towards 2000 bp (Fig. 3C, lane 6). This enhanced transformation probably reflects a combination of protection against intra- and extracellular nucleases and the ability for homologous recombination. That the transformation frequency decreases for smaller DNA fragments was already shown for the organisms *Acinetobacter calcoaceticus *and *Haemophilus influenzae*, especially beyond a minimal DNA size of 1 kb and 3.5 kb, respectively [23,24]. In the latter case this was explained by a partial degradation of 1.5 kb of the incoming transforming DNA before it gets integrated into the genome [24].

Another hypothesis that should be taken into consideration is the potential occurrence of uptake signal sequences (USS) in the gDNA samples versus the PCR derived fragments. Such sequences have been described for other gram-negative bacteria like *Neisseria gonorrhoeae *and *H. influenzae *[25,26] and it was shown that "the presence of a 10-bp uptake sequence enhanced a DNA fragment's ability to transform the gonococcus by four orders of magnitude" [27]. For *N. gonorrhoeae *and *H. influenzae *these sequences were estimated to occur every 1 kb [25] and 1248 bp [28], respectively, with a total number of 1465 copies of the USS (9-base pair in length) in the genome of *H. influenzae *Rd [28]. As the transformation frequencies of PCR-derived fragments were more than sufficient for the purpose of this study we did not follow up on the hypothesis of USS in *V. cholerae *and other *Vibrio *species but we will do so in the future.

A question we were often asked is "are there any special crab shells required for natural transformation to occur?". To circumvent the problem of acquiring crab shells we tested commercially available chitin sources including chitosan, chitin flakes and chitin powder. Except for chitosan we always got highly efficient natural transformation to occur.

Our final goal was to make use of a standard minimal medium instead of the complex defined artificial seawater medium. To boost the transformation efficiency we tested M9 minimal medium supplemented with four different salts/components: NaCl, HEPES, MgSO_4_C and CaCl_2_. As illustrated in Fig. 5 we saw significant positive effects after addition of Mg^2+ ^and/or Ca^2+^. Both of these cations were also shown to enhance natural transformation of *A. calcoaceticus *[19].

## Conclusion

We established an optimized procedure to genetically manipulate *V. cholerae *by chitin-induced natural competence (see Additional File 1 for a detailed protocol). The advantages of the new protocol are 1) its rapid feasibility (three days in total for the expedite version); 2) that PCR-derived donor DNA can be used given homologous flanking regions of at least 500 bp are present; 3) the chitin source is commercially available; 4) M9 minimal medium enriched for MgSO_4 _and CaCl_2 _can be utilized. Further studies will demonstrate whether other *Vibrio *species are also amenable to this new procedure.

## Authors' contributions

RLM contributed intellectually to this study, carried out experiments, and analyzed data. MB served as principal investigator, designed and coordinated the study, performed experiments, analyzed data, and wrote the manuscript. All authors read and approved the manuscript.

## Authors' information

RLM is a Master student at the Center for Systems Microbiology/Department of Systems Biology of the Technical University of Denmark. He performed a summer internship in the Blokesch lab at EPFL, Lausanne, Switzerland.

## Supplementary Material

Additional file 1 **This file provides a detailed natural transformation protocol based on the results obtained in this study**. Click here for file
