# Supplementary material for: Natural transformation of Vibrio cholerae as a tool - Optimizing the procedure
Source: BMC Microbiol. 2010 May 28;10:155. doi: 10.1186/1471-2180-10-155 (PMC2890613; doi:10.1186/1471-2180-10-155)
Supplement: Additional file 1 — This file provides a detailed natural transformation protocol based on the results obtained in this study. [file 1471-2180-10-155-S1.PDF]

Natural transformation of *Vibrio cholerae* as a tool – Optimizing the procedure

Rasmus L. Marvig and Melanie Blokesch

Transformation protocol

Day 1:

- Grow *Vibrio* strains, preferentially extracellular nucleases minus strains, to mid-log phase in LB. Wash and resuspend bacteria in 2 volumes of M9 minimal medium containing 5 mM $CaCl_2$ and 32 mM $MgSO_4$ and incubate them on chitin flakes for 16 hours. This will induce natural competence.
- On the same day prepare your PCR-fragment of interest containing a selective marker with ≥ 500 bp of homologous flanking regions on both ends. Purify the PCR fragment e.g. by using commercially available PCR purification kits.

Day 2:

- Exchange the medium against fresh supplemented M9 medium and add ≥ 200 ng of the prepared PCR fragment (higher amounts are recommended).
- Incubate for two hours.
- Detach bacteria by vigorously vortexing the chitin flakes.
- Plate bacteria on selective medium. To determine transformation frequencies plate cells from a dilution series on plain LB plates.

Day 3:

- Pick colony from selective plates and use for desired experiments.
- At that point, colony PCR as well as sequencing can be performed to confirm correct integration into the genome.
